# Detecting and Managing Mental Health Issues within Young Adults. A Systematic Review on College Counselling in Italy

**DOI:** 10.2174/1745017901713010061

**Published:** 2017-07-19

**Authors:** Alberto Ghilardi, Chiara Buizza, Egle Miriam Carobbio, Rossella Lusenti

**Affiliations:** Department of Clinical and Experimental Sciences, University of Brescia, Brescia, Italy

**Keywords:** College counselling, Psychological support, Students, Efficacy, Italy

## Abstract

**Background:**

College counselling can be considered as a front-line service in detecting and managing mental health issues within young adults. In this sense, it is important to investigate the effectiveness of counselling interventions.

**Objective:**

To provide a systematic review about college counselling in Italy; to assess which psychological interventions really meet student needs, and their effectiveness; to focus on the level of cohesion between Italian counselling services and the international guidelines about college counselling.

**Method:**

A systematic review about college counselling through PsycInfo and PubMed was carried out. Because of the scarceness of pertinent available articles, the survey was extended to Google Scholar and Riviste Web. Keywords: counselling, mental health, wellbeing, psychological support, university, students, Italy.

**Results:**

Out of thirty-four articles retrieved, 16 are relevant to academic counselling, the other 18 have been considered not pertinent to the aim of the present review. Data show a lack of homogeneity in methodology and organization between each University: different approaches towards students’ needs. Furthermore, no follow-up studies or measurement of effectiveness were found.

**Conclusion:**

This review is a contribution to disseminate the results of counselling experiences in Italy and represents an effort to encourage colleagues working in a web environment to share results and methods for a more organized protocol application.

## INTRODUCTION

Student’s mental health is a growing problem and an emergent topic of interest for psychologists and researchers. This issue has a worldwide resonance as the European Forum for Student Guidance (FEDORA) states [1, 2]. The number of university students with a serious mental illness has risen significantly both in severity and incidence over the past fifteen years [3, 4].

College is a critical period for all students: separation from parents, detachment from family, questions of purpose in life, orientation toward goals, new social relationships, new balance between work, study and private life, financial issues and higher studying demands are just some aspects in the development of a new identity. This process may bring about crises, anxiety and self-doubt. To cope positively with crises builds self-efficacy and self-esteem, even though it might temporarily jeopardize academic achievement. In the case of negative progression, identity development is shortened, resulting in pseudo-identity or identity diffusion and academic engagement is weakened. In general, this threshold means a deep reconsideration of complex developmental aspects in reaching adulthood [5]. This set of factors enhances the level of perceived stress: as a matter of fact, students suffering from mental disorders are mostly diagnosed with depression, anxiety and problems with self-worth regulation [6]. The American College Health Association [7] states that 90% of manifested diseases are psychological ones. A proportion of this might be due to more attention given to mental health in general, and more through diagnoses and treatment options. These explanations aside, however, there seems to be a real increase in the numbers of young people afflicted [8-11].

Taken all together, the need for an integrated learning support and mental health strategies is evident. Offering counselling to students is increasingly considered as a key academic service and can be seen as a front-line service in the detection and management of mental health issues in young adults at an early stage.

The introduction of counselling services in Italy only dates back to the 1980s, but the number of counselling centres has progressively increased over the last two decades. The first national recognition was in 2009, promoted by CRUI (Conference of University Rectors in Italy): 68 out of 80 universities, associated with CRUI, counted a counselling centre for students. But a second recognition has not yet been carried out.

This state of things does not offer a faithful image of the Italian psychological academic counselling situation [12].

Although in recent years the interest in college counselling has increased, Italian studies in this field are still very limited. Moreover, most of the studies available have incomplete outcomes, follow-up and study of efficacy. Italian literature is almost all descriptive: little attention is addressed to quantitative analysis [13]. The effectiveness of student counselling services has been a relatively neglected research area, only recently is this trend changing and some works focusing on this topic are circulating in Italy [14-16].

In North America, UK and USA, the scientific literature appears to be more homogenous. This trend is also due to official guidelines that refer to: operators structuring their interventions according to them [17-20]. In this way services and intervention outcomes would be comparable with each other’s. The criteria and guidelines consider the following areas: 1) counselling services’ relationship with the university college community; 2) counselling services’ functions and roles; 3) ethical standards; 4) counselling services’ personnel; 5) other related aspects such as economic resources, *etc*. [18].

Recently, the effort for providing official national guidelines in Italy has been headed by the Organization of Academic Psychology [21]. The goal is to define a global strategy and to consolidate clinical counselling tradition in the university field. This shared scheme of priority and practice aims to align operational models for a more homogenous and effective service targeted towards young people’s mental health. This also represents an effort to bring Italian reports to an international level of recognition.

The purpose of this systematic review, using Preferred Reporting Items for Systematic Reviews and Meta-Analyses - PRISMA guidelines [22], was: a) to assess which psychological interventions really meet student needs and their effectiveness; b) to focus on the level of cohesion between Italian counselling services and the international guidelines about college counselling [1, 18, 23]; c) and to assess whether these services are really effective. This is the first review about college counselling in Italy.

## METHOD

As mentioned above, the methodology of this study follows the PRISMA model [22]. A comprehensive literature search using PsycInfo and PubMed was conducted with no date restrictions to identify studies published on psychological counselling in Italian universities. The research excludes articles about career counselling and counselling for curricula orientation, even if they occur within a university context. Because of the paucity of studies, the research was extended to GoogleScholar and RivisteWeb. Moreover, it examined references from articles to identify further additional papers of interest. Keyword sequence options are the following: counselling, mental health, wellbeing, psychological support, university, student, Italy (both in Italian and in English). For reasons of clarity and organization, the review continues to collect articles for its own university.

The selection of the studies was conducted by two independent authors that sought a consensus in case of disagreement. To solve possible disagreements between the two experts, a third evaluator was requested for the due order.

## RESULTS

Thirty-four articles retrieved: 26 in Italian [4, 15, 24-47] and 8 in English [2, 12, 16, 48-52]. Of the total amount of 34 articles, only 16 give detailed descriptions of their academic counselling service, thus they have been considered relevant for the present review [4, 12, 15, 16, 27, 29, 31, 32, 37, 38, 44-46, 48-50].

The above articles refer to 8 specific Italian universities: Università degli Studi di Napoli Federico II, Università degli Studi di Milano-Bicocca, Università degli Studi di Padova, Università degli Studi dell’Aquila, Università degli Studi di Bologna, Università degli Studi di Bari, Università di Roma La Sapienza, Telematic University UniNettuno (Table **1**).

The other 18 articles have not been considered because they are partially incomplete: they respond to our specific keywords but they do not mention the tools and features of the academic counselling service offered. This is a clinical oversight that does not let us understand the psychological dimension of the counselling intervention. Indeed, some articles have been excluded because they describe other kinds of counselling consultation other than the clinical one, like job orientation or stress management programming.

Table **2** shows the data collected from the 16 articles taken into consideration: they are shown according to the five criteria of the international college counselling guidelines. Since there is no a proper correspondence between the structure of each article and the standards to which we refer: data have been inferred and reorganized. Articles have been gathered according to which university they refer to, and then compared with international parameters (Table **2**).

### Counselling Services Realtionship with the University Community

The first criterion refers to the location of the counselling centre within the university complex. This aspect is of interest because the accessibility, the familiarity and the strategic position of the setting enhances the probability of the service’s success. As a matter of fact, students need to feel that it is part of normal student life and on the same level and worthy of other services promoted by the university. This neutral and ergonomic aspect of the setting lessens the resistance in using the service and the stigma of a psychological, mental support. The presence of a psychological centre within the university’s confines, promotes the role and the responsibility of the university’s community in supporting a wider wellbeing.

From data collected we can infer that most counselling services for students take place inside university buildings. The service is run either by a single group of professors [31, 46] or by the college clinical hospital [49].

The articles considered do not report any details about the internal settings or other specifications about space layout for clinical colloquia and interventions. We have little information about the network surrounding the psychological service: only two articles mention a collaboration between the university and the local healthcare public service agency or other organisation for the right to learn [12, 38]. It is also relevant that no connections between internal university services are mentioned.

## Counselling Services Functions and Roles

The second criterion refers to features and roles of the counselling services offered (career counselling, psychological counselling). In more detail, there are three main aspects of a counselling service: the first is to support students who experience psychological difficulties; the second is to help students improve their life skills; the third is to promote students’ wellness.

This review shows each university’s counselling has different psychological attitudes, which lead to different ways of putting them into practice: the main approaches are humanistic, psychoanalytic, psychodynamic and cognitive-behavioural.

Generally, the interventions are short (they last from two to six meetings); in some cases there is the possibility to proceed with psychotherapy interventions directly after the psychological one, either through university services or through other public and private institutions.

Counselling interventions last between 40 and 90 minutes. Each service guarantees free access to students; they can contact the service by telephone or by email; the waiting time from the first contact and the feedback for scheduling the appointment is a week (seven working days). Five of the eight services considered only offer services for single student meetings, the other three offer group meetings for students with similar difficulties. There are two unique cases where one university opens its service to the young community outside of the university’s confines [38] and another one gives counselling services just to students of medicine [44].

All services have specific areas for their activities and specific instruments to register data and results. All services provide students with a socio-demographic data sheet: the variables collected concern age, marital status and gender, academic career, geographic origin, residence (distance between home and university, how they arrive at the university, where they live, *etc*.), other contacts with psychological services, reasons behind accessing the service.

Standardized assessment instruments are reported in only eight articles (7 different universities) [4, 14-16, 37, 44, 45, 50]. In the other articles the authors use non-standardized instruments such as ad hoc questionnaire scales. Only two articles mention a scale for the assessment of satisfaction [4, 44]. The services guarantee anonymity and privacy through a specific form of informed consent.

The guidelines emphasize the importance of each service to engage in research in order to evaluate its own operation. Unfortunately, only two universities present data reporting the effectiveness of their services [15, 16].

## Ethical Standards

The third criterion refers to the ethical and legal standards that a counselling service guarantees, with particular reference to privacy and the observance of the code of practice.

Articles provide information about the careful training of the counsellor, the regular team meetings and the discretion of personal information, in order to provide the best consultation possible, tailored to each student.

## Counselling Services’ Personnel

The fourth criterion is about the characteristics expected from the counselling service’s personnel. Staff members should have an appropriate academic degree and they should maintain a continuous training program, including presentations, research reports, discussion and so on. Moreover, a staff director should supervise service activities and manage the budget. Seven services have a staff comprising just professionals with a psychotherapeutic profile, in all the other centres there is a psychologist and new graduate students. The staff has appropriate coursework and training in psychological assessment; there is always a service director to supervise the staff work.

## Related Guidelines

The fifth criterion refers to indispensable counselling service aspects: staff members should be encouraged and supported in accepting leadership responsibilities within their respective local and national organizations and they should train continuously. It is also important that the staff participate in community activities related to their profession. Data collected do not allow evaluation of this last criterion’s services because of a lack of information.

## DISCUSSION

Despite all authors declaring a commonality of purpose in structuring the psychological support services, there is a broad heterogeneity in published contents and services offered. From data collected it is not possible to find a common structure for interventions and therapeutic tools used. It is possible that this broad heterogeneity stems from several reasons: from the different theoretical approaches the counselling service is based on, to the socio-cultural environments in which it is set and to the specific internal organization of the university. Moreover, there is no national legislation: the launch of these services is the individual university’s choice.

At this time, there are few studies about the effectiveness of these services and there are no follow-ups measuring the long-term effect of the consulting service.

From data collected, some common elements in all the counselling experiences analysed emerge: they are all free services, they all offer a brief intervention to actively support students and their wellbeing and all the services guarantee privacy and discretion. The professional staff focuses on the students’ difficulties, help them in becoming more self-aware and in developing coping strategies. Moreover, all the articles show how important teamwork is: a network of professionals seems to be more useful and influential in tailoring the best response to each student’s problem.

What is evident in this review is a strong theoretical literature that does not always reflect a quantitative analysis about data presented [13]. Despite studies on the effectiveness of counselling services, there have been areas of research neglected until now, but it seems that the trend is gradually changing and it is even more important to assess the quality of interventions [16].

The few data collected show that the students who have followed a counselling path improved their welfare, experienced a strengthening of their ego, a more positive self-perception and more sureness [15], as well as a reduction in the level of distress and psychopathological symptoms [16].

Psychological intervention seems to promote changes that impact positively on the quality of students’ life: after a few meetings, they have more confidence in themselves, tolerate their fears better, strengthen their relationships and goals [53]. Generally, psychological support seems to positively influence both the personal sphere and academic performance [54-56].

Despite the positive evaluation of the students who use these services, counselling is not the main resource for the students when they have some difficulties. Generally, the affluence of students to counselling services is only 2-4% of the total student population, with a slight increase in women [2, 9, 57, 58].

You may cite different reasons associated with the underutilization of these services, among these the most important are the students’ tendency to underestimate their difficulties [57, 59], the fear of being stigmatized [60, 61], and the lack of knowledge of the counselling services [10].

Other studies have also shown that students from ethnic minorities, foreign students and students with disabilities are reluctant to use these services [58, 62]. On the other hand, factors that seem to favour access to counselling services are to have had previous experience with mental health professionals and the presence of personal characteristics such as a good ability to recognize emotions and optimism for the future [63].

It is believed that the presence of a counselling service within universities is really essential for the delicate evolutionary phase that students go through. Moreover, it is important to remember that most mental diseases have their onset between the ages of 18 and 24 [64, 65]. Thus, college counselling could become an important front-line service in detecting and managing mental health issues within young adults. Epidemiological data show that people with a mental disorder come for treatment some years after onset, when symptoms have stabilized but interfere with the ability to perform expected social roles, thus, resulting in a chronic disorder [66-68]. Nevertheless, very few college counselling services collaborate with Departments of Mental Health (DMHs). This collaboration could be very helpful to young people in offering early access to effective treatments, to counter the widespread belief that mental health services are dedicated to chronic patients with a high level of psychosocial disability and high care needs, and to fight the stigma that accompanies people who suffer from mental disorders, given that they play a key role in influencing levels of information, awareness, treatment, and prevention [69, 70].

It is believed that only through a greater cooperation between counselling services and other bureaus (both academic and non- academic such as mental health services) in order to respond in a more integrated way to the different needs of students, and through scientific studies that evaluate the effectiveness of these services, will it be possible, in Italy, for counselling to become an integral part of the evolutionary and education process of university students, as stated in the guidelines on college counselling of the American Psychological Association [17].

## Figures and Tables

**Table 1:** Detailed of reviewed articles about Italian college counselling.

**Author**	**Sample** **(n)**	**Age Range** **Mean Age (sd)**	**Number of Meetings**	**Approach**	**Tools**
Adamo *et al.* [12]	72	19-44 yr 22.0 (--)	4	Psychoanalytic	NA
Adamo *et al.* [27]	100	NA	4	Psychoanalytic	Detailed Protocol Based on the Record of the Interview
Adamo *et al.* [48]	NA	NA	4+Follow-up	Psychoanalytic	NA
Cecchini & Langher [32]	Case Report	22 yr	5+Follow-up	Psychodynamic	Personal Data Sheet
Cimino *et al.* [50]	32	-- 40.8 (10.9)	NA	Psychodynamic	Adult Self Report
Ciuffo & Farnese [31]	50	18-41 yr 24.6 (--)	6	NA	NA
Dazzi *et al.* [4]	89	18-45 yr 24.4 (5.1)	4+Follow-up	Psychodynamic	Adult Self Report
De Lauretis *et al.* [45]	47	-- 24.8 (5.9)	3	CBT	GHQ SCL-90 Depression Rating Scale Brief Cope
Giusberti & Bertolini [38]	2.024 (open also to young people not student)	19-43 yr --	4	NA	Motivational and Autobiographic Questionnaire
Laquale *et al.* [15]	79	18-34 yr 23.8 (3.4)	5+Follow-up	Psychodynamic	Adjective Check List
Lia *et al.* [44]	44	21-35 yr 25.0 (3.4)	4+Follow-up	NA	SCID-I Clinical Version
Maccarone & Zanasi [29]	100	20-25 yr --	2	NA	Scale of Emotion (not standardized)
Marini *et al.* [37]	118	19-33 yr 24.4 (2.8)	3+8	Psychodynamic	MiniPLUS SCID II
Monti *et al.* [46]	726	19-47 yr 24.7 (3.4)	4	Humanistic with integrative model	Global Functioning Evaluation
Strepparava *et al.* [16]	45	20-37 yr 23.8 (4.3)	4 (to maximum of 10)	Cognitive	CORE-OM SCL 90-R ERQ
Valerio & Adamo [49]	Case Report	NA	4+Follow-up	Psychoanalytic	NA

(NA) Data non available

**Table 2:** Cohesion between Italian counselling services and the international guidelines.

**University**	**Author**	**Collocation/** **relations**	**Services offered**	**Ethical Standards**	**Staff**	**Other Aspects**
L’Aquila	De Lauretis *et al.* [45]	NA	Individual and group counselling	Yes	Counsellor, graduate students	Free Access Internal Economic Resources
Bari	Laquale *et al.* [15]	NA	Individual counselling	Yes	Psychotherapist, supervision group	Free Access Internal Economic Resources
Bologna	Giusberti & Bertolini [38]; Monti *et al.* [46]	Local Health Authority	Individual and group counselling, psychotherapy	Yes	Psychotherapist, graduate students, teachers, director	Free Access Collaboration with the NHS
Milano Bicocca	Adamo *et al.* [12]; Strepparava *et al.* [16]	National Health Service	Individual counselling	Yes	Psychotherapist, director	Free Access Collaboration with the NHS
Napoli	Adamo *et al.* [27]; Adamo *et al.* [48]; Valerio & Adamo [49]	Regional Office of Students	Individual counselling	Yes	Psychotherapist, psychologist	Free Access Grants from the Regional Office of Students
Padova	Maccarone & Zanasi [29]; Marini *et al.* [37]	Italian Institute for the Right to Education	Individual and group counselling, psychotherapy	Yes	Teachers, psychiatrist, psychologist, graduate students	Free Access Economic Resources: after the first 3 free talks, 8 interviews will be proposed, at a cost of 35 euros each one
Roma	Lia *et al.* [44]; Dazzi *et al.* [4]; Cecchini & Langher [32]; Ciuffo & Farnese [31]	Laziodisu (Center of research)	Individual counselling	Yes	Psychologist, psychotherapist, graduate students, teachers, physician, psychiatrist	Free Access
Uninettuno	Cimino *et al.* [50]	NA	Individual counselling	Yes	NA	NA

(NA) Data non available

(NHS) National Health Service
